# Evaluating Medical Education Escape Rooms: A Scoping Review Using the Kirpatrick Model

**DOI:** 10.12688/mep.21095.3

**Published:** 2026-01-29

**Authors:** Hannah Sturm, Garrett Brown, Eric A. Gantwerker

**Affiliations:** 1 Zucker School of Medicine at Hofstra/Northwell, Hempstead, New York, USA; 2 Feinstein Institutes for Medical Research, Manhasset, New York, USA; 3 Department of Otolaryngology, Northwell Health, New Hyde Park, New York, USA; 4 Division of Pediatric Otolaryngology, Cohen Children’s Medical Center, New Hyde Park, New York, 11040, USA

**Keywords:** Escape Rooms, Kirpatrick Model, Simulation-Based Learning, Gamification, Team-Based Learning

## Abstract

**Background:**

Medical educators are increasingly exploring innovative strategies such as medical education escape rooms (MEERs), team-based games in which participants solve puzzles and complete tasks within a time limit to achieve a goal, to enhance learner engagement. This scoping review aimed to evaluate the utility of MEERs as a teaching method in medical education.

**Methods:**

A scoping review was conducted using PubMed, MedEdPORTAL, and Scopus. Inclusion criteria were studies published in the past 12 years, written in English, involving medical students and/or residents, and reporting measurable outcomes. Exclusion criteria included systematic reviews, proof-of-concept studies, and studies outside medical school or residency contexts. Titles and abstracts were screened, followed by full-text review by two independent reviewers (HS and GB). A total of 20 studies met inclusion criteria. Data were extracted and analyzed for study characteristics and outcomes.

**Results:**

All 20 studies assessed student reactions, with overwhelmingly positive responses. Reported outcomes included increased engagement, satisfaction, enjoyment, perceived learning, improved teamwork, clinical relevance, desire for more MEERs, and recommendations to peers. Nine studies (45%) evaluated learning outcomes, with significant improvements in pre- to posttest scores. MEERs were found to be more effective than flipped classrooms and traditional lectures and equally effective as case-based learning. One study evaluated behavioral change but found no significant impact on long-term behavioral outcomes.

**Conclusions:**

Escape rooms are an effective and engaging educational strategy in medical education, supporting knowledge and skill acquisition. Further research is needed to evaluate their long-term behavioral impact in clinical settings.

## Introduction

Medical educators are increasingly adopting innovative strategies to enhance learner engagement in both medical school and residency programs. One such strategy is gamification, the use of game elements in non-game contexts, which has been shown to improve motivation, satisfaction, and academic performance among health professions learners ^1^. In contrast, serious games are structured, goal-oriented game experiences intentionally designed for purposes beyond entertainment, most commonly to teach, train, or change behavior, by immersing players in interactive, often realistic scenarios that promote active learning, decision-making, and skill development ^2^.

A hybrid of these two approaches is the Medical Education Escape Room (MEER), a team-based game in which players solve puzzles and complete tasks under time constraints. In medical education, MEERs have been implemented in diverse formats to suit various learning objectives. Some MEERs are designed as serious games, immersing learners in realistic, high-pressure clinical scenarios such as triage, infection control, and emergency response, thereby fostering critical thinking and teamwork under stress ^3, 4^. Others adopt a gamification approach, incorporating puzzles and challenges into classroom or simulation settings to reinforce curricular content through game mechanics, such as point systems, collaboration, and immediate feedback ^5, 6^. Many MEERs integrate elements of both approaches, creating hybrid designs that combine immersive narrative scenarios with features such as time constraints, leaderboards, and competitive scoring to enhance learner engagement and motivation ^7^.

They have been used for purposes such as identifying and reporting safety hazards, setting patient safety priorities, assessing topic-specific knowledge, teaching clinical skills, improving teamwork, promoting well-being, and orienting new learners to clinical environments. MEERs differ from general educational escape rooms in that they are grounded in clinical reasoning and patient safety contexts, requiring the integration of medical knowledge, teamwork under time pressure, and decision-making aligned with professional competencies. While educational escape rooms in other domains may focus on concept reinforcement or engagement, MEERs simulate authentic healthcare environments and align explicitly with competency-based medical education frameworks.

These tools have been used to teach both content knowledge and non-cognitive skills such as teamwork and communication ^4^. MEERs have also been adapted for various platforms, including in-person, virtual, and hybrid environments, demonstrating flexibility in curricular integration. They have been used for purposes such as identifying and reporting safety hazards and setting patient safety priorities ^8, 9^, assessing topic-specific knowledge ^10^, teaching clinical skills ^11^, improving teamwork ^12^, promoting well-being ^13^, and orienting new learners to clinical environments ^14^.

MEERs align with adult learning and experiential learning theories by offering immersive, learner-centered experiences that promote higher-order thinking, skill development, and knowledge application ^15^. Their gamified nature fosters intrinsic motivation and learner engagement, while the team-based format provides opportunities for peer learning and collaborative problem-solving in a low-risk setting.

To evaluate the effectiveness of such interventions, the Kirkpatrick Model provides a widely used framework encompassing four levels: reaction, learning, behavior, and results. *Reaction* assesses learner satisfaction and perceived value through post-activity surveys. *Learning* measures knowledge or skill acquisition, typically using pre- and post-tests. *Behavior* examines the application of learned skills in real-world settings, assessed through observation or follow-up surveys. *Results* evaluate the broader impact of the intervention on outcomes or institutional goals ^16^.

This study aimed to evaluate the utility of MEERs as a didactic tool in medical education through a scoping review. A preliminary search revealed no existing scoping reviews on this topic, highlighting a gap in the literature and an opportunity to synthesize and evaluate current practices, outcomes, and future directions for this emerging instructional strategy.

## Materials and methods

### Overview

The utility of MEERs in medical education was explored using a scoping review based on the methodology described by Arksey and O’Malley ^17^. A scoping review protocol was written to describe the search strategy and is available from the corresponding author upon request. This paper adheres to the JBI Scoping Review Network recommendations for the extraction, analysis, and presentation of results ^18^. The scoping review was performed in November 2022. IRB approval was not necessary for this study as there was no inclusion of human subjects. The review protocol was registered on the Open Science Framework to enhance transparency and methodological rigor.

### Inclusion criteria

To be included, original peer-reviewed articles had to meet four criteria. First, the article had to be published in the last 12 years (2010 – 2022). Second, the first version of the article needed to be in English. Third, the study population had to include medical students and/or residents in an accredited medical school or residency program either inside or outside the United States. Fourth, the study needed to have measured outcomes. Exclusion criteria included systematic reviews, proof-of-concept studies, and studies conducted in a healthcare discipline outside of medical school or residency.

### Search strategy

The initial limited search of databases related to the use of MEERs in medical education showed that the databases PubMed, MedEdPORTAL, and Scopus were the most relevant to the topic and text words contained in the titles and abstracts and index terms were collected to describe the articles including “escape room”, “breakout room”, “breakout box,” “puzzle-based learning,” and “gamification”. Initial searches across all three databases showed that the term “breakout room” mostly applied to Zoom breakout rooms, while “breakout box” and “puzzle-based learning” often describe recreational or informal activities outside the context of structured educational interventions. Similarly, “gamification” was too broad, yielding many studies unrelated to escape room-based learning. Based on these findings and the consistent use of the term “escape room” in recent literature to describe educational escape room activities, the final search strategy was restricted to this term to ensure precision and relevance. Therefore, a second search using the search term “escape room” was conducted across the three included databases. Then, a reference list of all identified reports and articles was created. All identified articles from the searches were transferred to an online systematic review software for screenings (Covidence systematic review software, Veritas Health Innovation, Melbourne, Australia). The reviewers did not contact authors of primary studies or reviews for further information.

### Selection of relevant and reliable studies

**Figure 1.**
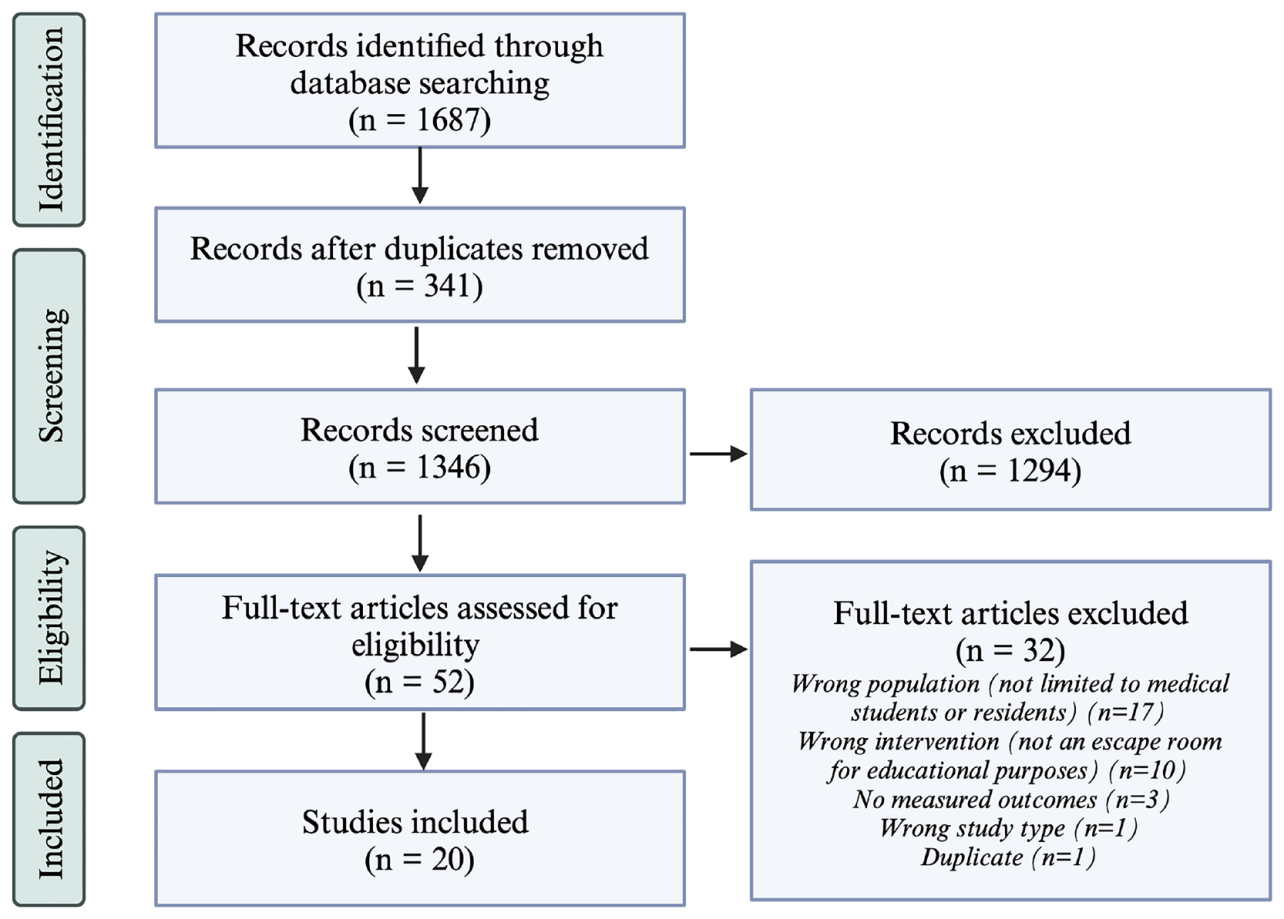
PRISMA Flow Diagram for the Scoping Review Process. The number of articles included after each step of the scoping review process is listed in parenthesis. On the right, the number of excluded articles is listed as well as the reasoning for article exclusion after full-text review. This figure was created using BioRender ^TM^.

By applying the inclusion criteria, two reviewers (HS and GB) screened the articles on the reference list for selection ( Figure 1). The first screening was based on the title and abstract only and the second on a full-text review. All conflicts between the two reviewers that arose during the screening stages were discussed until an agreement was reached. If needed, a third reviewer was consulted to reach a consensus on including that article.

### Extraction of the results

After the articles were selected from the second full-text screening, data was recorded in a Microsoft Excel spreadsheet. Variables collected included: author(s), year of publication, country of study, study aims or purpose, population and sample size, methodology, type and duration of the MEER, format (virtual or in-person), outcomes measured, and key findings. Outcomes and key findings were categorized using the Kirkpatrick Model of Training Evaluation, which was selected as the guiding framework due to its broad acceptance and systematic approach to classifying educational outcomes. Although most included studies assessed Level 1 and Level 2 outcomes, the framework offered a consistent structure for organizing and comparing findings across diverse study designs. The Kirkpatrick Model describes four levels of educational value: Reaction, Learning, Behavior, and Results.

Kirkpatrick Level 1 (Reaction) outcomes encompassed learners’ engagement, enjoyment, satisfaction, perceived clinical relevance, self-reported knowledge gain, desire for greater integration of MEERs into curricula, willingness to recommend MEERs to others, improvement in teamwork, and enhancements in workplace social capital (defined as the strength of social connections and trust within a professional setting). Kirkpatrick Level 2 (Learning) outcomes included performance on pre- and post-intervention assessments and comparisons to outcomes from traditional instructional methods. Kirkpatrick Level 3 (Behavior) outcomes addressed observed or self-reported behavioral changes following MEER participation ^16^.

### Data analysis

Descriptive analysis of study characteristics and outcome measures was conducted using Microsoft Excel. For studies that reported the proportion of students endorsing specific outcome measures, the percentage of agreement and the corresponding sample size were recorded. For studies utilizing Likert scales (typically 5-point scales ranging from “strongly disagree” to “strongly agree”), the mean score and sample size were documented.

When reported, Cohen’s *d* values were extracted directly from the original studies. When not explicitly reported, Cohen’s *d* was estimated for pre- and post-intervention outcomes using a within-subjects (paired) approach when sufficient information was available. In cases where only summary statistics were reported, effect sizes were approximated using the standardized mean difference based on pooled standard deviations. These approaches are consistent with established methodological guidance for estimating effect sizes from pre–post study designs ^19^.

## Results

### Study selection

The initial search yielded 1,687 articles. After removing duplicates, 1,346 articles were screened by title and abstract by two independent reviewers (HS and GB), resulting in the exclusion of 1,294 articles. The remaining 52 articles underwent full-text screening, and 32 were subsequently excluded. A total of 20 articles met the inclusion criteria and were included in the final analysis and data extraction.

### Article characteristics

Of the 20 included studies, 13 were conducted in the United States. Four studies evaluated MEERs implemented through virtual platforms. The study populations included residents in ten studies, medical students in nine, and both groups in one study. The publication years ranged from 2018 to 2022. The average number of participants per study was 73.1, and the average duration of the MEER activity was 43 minutes ( Table 1).

**Table 1.** Characteristics, Methods, and Outcomes of Scoping Review Articles. The mean percentage of medical students or residents who agreed with each outcome is reported for individual studies. Some studies used a 5-point Likert scale, where 1 indicated “strongly disagree” and 5 indicated “strongly agree.” Studies that included pre- and post-survey data are labeled “Pre” and “Post,” with the type of survey noted above each entry. For clarity, in Dimeo *et al*., the survey sites were abbreviated as University of California–Irvine (UCI) and Prisma Health Upstate (Prisma) in the table.

Review	Aims	Escape Room Design	Study Sample	Sample Size	Outcomes
Cantwell *et al*., 2022 ^20^	Determine attitudes of students towards gamification, compare the virtual ER to a flipped classroom format.	Virtual, serious game, gamification elements	3 ^rd^ and 4 ^th^ year medical students	134	Engagement: 86%, Satisfaction: 81% Clinical relevance: 78% Desire for more: 74%
Cates *et al*., 2020 ^10^	Test knowledge of toxicologic ingestion and antidotes.	Virtual, serious game, gamification elements	Residents	46	Clinical relevance: 54% Perceived learning: 62.5% Recommend to others: 70.5%
Diemer *et al*., 2019 ^8^	Educate interns on the program’s local safety priorities and use of the voluntary reporting system.	In person, serious game, gamification elements	Interns	120	Confidence identifying safety hazards: Pre 6.35/10 → Post 8/10 ( *p* < 0.001) Effect size: *d* ≈ 0.8–1.1
Dimeo *et al*., 2022 ^21^	Determine the value of virtual ERs compared to didactic lectures on infectious disease.	Virtual, gamification only	Residents	30	Enjoyment: 78.6% Knowledge assessment: UCI: Pre 77.8% → Post 88.9% Effect size: *d* = 1.25 Prisma: Pre 73.81% → Post 89.68% Effect size: *d* = 1.36
Donovan *et al*., 2021 ^22^	Assess the feasibility of teaching a critical care simulation scenario through an interactive online game.	Virtual, serious game, gamification elements	3 ^rd^ year medical students	66	Enjoyment: 92.4% Desire for more: 90.9% Perceived learning: 93.9–97%
Jaffe *et al*., 2021 ^9^	Educate interns on identifying and mitigating safety hazards aligned with institutional priorities and reporting safety events.	In person, serious game with gamification elements	Interns	120	Self-reported comfort identifying patient-safety hazards: Pre 6.3/10 (SD = 1.57) → Post 8/10 (SD = 1.20) ( *p* < 0.001) Effect size: *d* = 1.22
Khanna *et al*., 2021 ^23^	Determine the feasibility of an ER as a learner-centered model for reinforcing knowledge and promoting team-building skills.	In-person, gamification only	Internal medicine residents	86	Engagement: 4.57/5 Enjoyment: 4.89/5 Satisfaction: 4.89/5 Clinical relevance: 3.78/5 Improving teamwork: 4.48/5 Desire for more: 4.38/5 Recommend to others: 4.74/5
Lundholm *et al*., 2022 ^13^	Evaluate the effect of a medical ER on intern residents’ workplace social capital scores.	In person, serious game, gamification elements	Internal medicine interns	52	Workplace Social Capital Pre 96% → 100% post ( *p* = 0.003)
Martin & Gibbs (2022) ^14^	Explore the use of ERs in exposing students to the simulated patient environment.	In person, serious game, gamification elements	Preclinical medical students	148	Perceived learning: 4.0/5 Clinical relevance: 82%
Podlog *et al*., 2019 ^11^	Teach core emergency medicine content and procedural skills, enhance team-building skills.	In person, serious game, gamification elements	Medical students and residents	30	Clinical relevance: 94% Perceived learning: 82% Desire for more: 100%
Zhang *et al*., 2019 ^24^	Enhance active learning and engagement with voluntary event reporting education.	In person, serious game, gamification elements	Interns	130	Engagement: 41.18%
Guckian *et al*., 2020 ^25^	Determine the impact of ERs on student perceptions of dermatology.	In person, gamification only	3 ^rd^ year medical students	101	Enjoyment: 100% Improved teamwork: 100% Confidence identifying skin conditions: Pre 31.3% → Post 81.3% Confidence performing exams: Pre 12.5% → Post 68.8%
Faysal *et al*., 2022 ^26^	Evaluate the effectiveness of ERs vs. case-based learning in clinical dermatology.	In person, gamification only	4 ^th^ year medical students	97	Knowledge assessment: Pre 3.7/8 → Post 7.3/8 (p = 0.000) Effect size: *d* = 3.60
Liu *et al*., 2020 ^27^	Evaluate participant satisfaction and learning through a pediatric radiology themed ER.	In person, gamification only	Undergraduate medical students	19	Enjoyment: 94% Desire for more: 89.5%
Akatsu *et al*., 2022 ^28^	Determine the educational value of ERs as part of the final assessment for a medical school course.	In person, serious game features only	1 ^st^ year medical students	140	Confidence in physical examination skills: Pre 49% → Post 73%
Kinio *et al*., 2018 ^5^	Assess the effect of a vascular surgery themed ER on student satisfaction, motivation, and engagement.	In person, serious game, gamification elements	1 ^st^ and 2 ^nd^ year medical students	13	Enjoyment: 100% Improved teamwork: 100% Perceived learning: 75%
Backhouse & Malik (2018) ^29^	Use gamification to conduct a patient safety simulation for medical students.	In person, serious game, gamification elements	3 ^rd^ year medical students	19	Clinical relevance: 100% Perceived learning: 100%
Rosenkrantz *et al*., 2019 ^30^	Determine the use of an ER for education at the emergency medicine summer school.	In person, Gamification only	Medical students	49	Engagement: 78% Enjoyment: 98% Recommend to others: 85%
Jambhekar *et al*., 2019 ^31^	Teach radiology content and enhance team building and perseverance.	In person, serious game, gamification elements	Residents	144	Enjoyment: 4.85/5 Satisfaction: 4.85/5
Zhang *et al*., 2018 ^12^	Determine the use of ERs to enhance team-building skills.	In person, gamification only	Residents, one faculty member	10	Enjoyment: 100% Clinical relevance: 100% Recommend to others: 100% Improved teamwork: 90%

### Purpose of escape room

The MEERs incorporated a range of game elements. Twelve studies implemented serious games with gamification components, seven used gamification elements only, and one used serious game elements exclusively. The number of puzzles per MEER ranged from 3 to 17, and nine studies allowed participants to receive hints to help progress through the activity. The MEERs addressed various educational goals: ten studies focused on reinforcing or assessing clinical knowledge and skills, five aimed to teach new content through team-based gamified learning, three emphasized safety education and hazard mitigation in clinical settings, and one focused primarily on team bonding.

### Kirkpatrick model classification of training education

All studies evaluated the feasibility of MEERs using Kirkpatrick Level 1 outcomes, which assess learner reactions. In 45% of the studies, clinical relevance was examined, with 54–100% of participants agreeing that ERs were relevant to clinical practice (average Likert score: 3.78/5). Enjoyment was assessed in 45% of studies, with 92.4–100% of participants reporting enjoyment (average Likert score range: 4.85–4.89). Perceived learning was evaluated in 35% of studies, with 62.5–100% of learners indicating knowledge gain (average score: 4/5). Teamwork was assessed in 35% of studies, with 90–100% of participants reporting improved teamwork skills (average score: 4.48). Engagement was evaluated in 30% of studies, with 41.18–86% of participants feeling engaged (average score: 4.57). In 25% of the studies, desire for more MEERs in the curriculum was assessed, with 74–100% of learners expressing interest in additional sessions (average score: 4.38). In 20% of studies, participants were asked if they would recommend the MEER to colleagues; 70.5–85% responded affirmatively (average score: 4.74). Satisfaction was assessed in 15% of studies, with 81% of learners reporting satisfaction (average score range: 4.85–4.89). One study also measured workplace well-being before and after the MEER, showing an increase in workplace social capital from 96% to 100% (p = 0.003) ^13^ ( Table 1).

**Figure 2.**
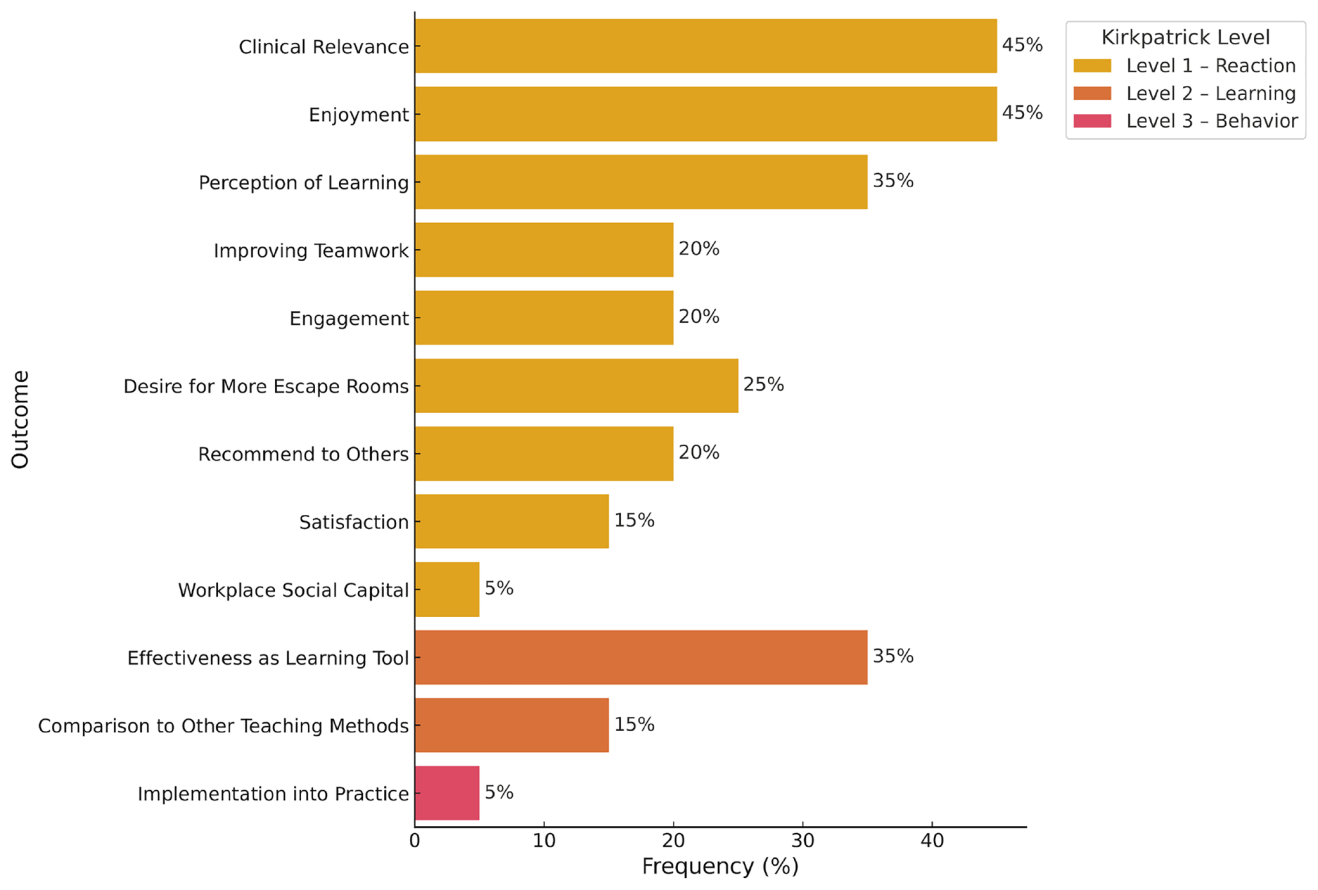
Escape Room Outcomes Based on Kirpatrick Levels of Education. Level 1 Outcomes are highlighted in yellow, Level 2 Outcomes are heighted in orange, and Level 3 Outcomes are highlighted in red. The percentage of articles discussing the outcome of interest is shown on the X axis. Note: Workplace Social Capital Score is a measurement of resident well-being. This figure was created by executing Python code in Google Colab ^TM^.

Nine studies (45%) evaluated Kirkpatrick Level 2 outcomes, which assess learning through pre- and post-intervention measures. Seven of these studies (35%) reported improvements in participant scores following the MEER activity ( Figure 2). For example, in studies focused on safety hazard identification and reporting, mean confidence scores on a 10-point scale improved from 6.35 to 8.00 post-session ( *p* < 0.001), corresponding to a large estimated effect size (Cohen’s *d* ≈ 0.8–1.1) ^8^. Another study reported a comparable increase in mean confidence scores from 6.3 to 8.0 on a 10-point scale (p < 0.001), with a large effect size (Cohen’s *d* = 1.22), indicating a substantial improvement following the intervention ^9^. Dimeo et al. reported that residents at two programs demonstrated significant learning gains: University of California–Irvine residents improved from 77.8% to 88.9% ( *p* = 0.028; Cohen’s *d* = 1.25), and Prisma Health Upstate residents improved from 73.81% to 89.68% ( *p* = 0.002; Cohen’s *d* = 1.36) ^20^. Faysal et al. documented a score increase from 58% to 86% (p = 0.000), assessing both immediate learning and retention after two weeks. Pre-test scores of 3.7/8 (range 2–6) increased to 7.3/8 (range 4–8) immediately after the session and remained at 7.3 (range 5–8) two weeks later, corresponding to very large effect sizes for both immediate ( *d* = 3.60) and two-week retention ( *d* = 2.72) ^21^. A dermatology-focused study similarly demonstrated an increase in confidence identifying skin conditions from 31.3% to 81.3% and in performing systematic skin examinations from 12.5% to 68.8% ^22^.

Fifteen percent of the studies compared MEERs to traditional teaching methods such as flipped classrooms or case-based learning (CBL). These comparisons often showed greater improvements in learner performance with MEERs. For instance, Cantwell *et al.* found that average Likert scores for content clarity and effectiveness of educational materials in the MEER (3.6 ± 0.63 and 3.6 ± 0.63 respectively) were rated higher than in flipped classroom sessions on chest pain (3.5 ± 0.67 and 3.4 ± 0.77 respectively for the two categories) and abdominal pain (3.2 ± 0.77, 3.1 ± 0.80 respectively) (p = 0.0491; p<0.001) ^20^. Similar trends were observed in residency programs, where MEER participation led to improved quiz scores, while lecture-based activities did not yield significant gains ^21^. One study comparing MEERs with CBL found both methods equally effective, with a slightly higher improvement between pre and post test scores in the MEER group (48.3%) compared to the CBL group (48.2%) ^26^.

Only one study assessed Kirkpatrick Level 3 outcomes, which evaluate behavioral change. This study aimed to improve residents' understanding of patient safety priorities and voluntary event reporting systems. Six months after the intervention, there was no significant increase in the use of the reporting system compared to historical controls, indicating no measurable behavioral change ^9^. No studies assessed Kirkpatrick Level 4 outcomes, such as reductions in patient safety hazards as a result of the training.

## Discussion

The use of MEERs has proven to be an engaging, motivating, and effective strategy in medical education. This scoping review shows that MEERs are consistently associated with positive learner reactions and educational outcomes among medical students and residents worldwide. Studies reported that learners were engaged, satisfied, and enjoyed the activity. Participants also found the experience relevant to their clinical training, gained knowledge and teamwork skills, and expressed interest in incorporating more MEERs into their curriculum. Many even recommended the activity to peers. Positive emotional responses such as enjoyment and enthusiasm are linked to academic success and improved cognitive processing ^32^, reinforcing the value of using MEERs as a tool to enhance learning and student well-being.

This scoping review illustrates that MEERs are not only consistently well-received but also demonstrate measurable educational benefits across multiple levels of the Kirkpatrick framework. Learner engagement, satisfaction, and enjoyment were near-universal themes, suggesting that MEERs are particularly effective in fostering positive emotional responses that are known to enhance motivation, cognitive processing, and academic success. These findings support the role of MEERs as a powerful pedagogical tool for enhancing the learner experience in both medical school and residency contexts.

Importantly, the review reveals that MEERs are not just entertaining games, but they are educationally effective. Nearly half of the included studies demonstrated significant improvements in knowledge or confidence through pre- and post-intervention assessments. These findings align with results from other health professions disciplines such as pharmacy and nursing ^33^, reinforcing the broader applicability of escape rooms as an interdisciplinary teaching modality. MEERs appear especially well-suited for reinforcing prior knowledge, clinical protocols, and team-based decision-making, areas where active learning and immersive environments offer distinct advantages over passive instruction.

Comparative studies further underscore the value of MEERs. When measured against flipped classrooms and traditional lectures, MEERs often yielded greater knowledge gains and learner satisfaction. This suggests that the integration of gamified and experiential elements may be more effective at stimulating critical thinking and long-term retention than conventional didactic approaches, which is supported by current educational theories supporting active learning ^34^. However, when compared to case-based learning, MEERs performed similarly, suggesting they may function best as complementary tools rather than outright replacements for traditional methods.

Despite their strengths, MEERs have not yet demonstrated consistent impact on behavior change (Kirkpatrick Level 3) or outcomes in clinical practice (Level 4). Only one study attempted to measure behavioral change, and it found no significant improvement in event reporting behaviors post-intervention. This highlights a critical limitation of MEERs. While they effectively influence short-term learning outcomes, translating this knowledge into sustained behavioral change likely requires more than a single educational encounter. Structural, cultural, and institutional barriers, such as hierarchical team dynamics, limited reinforcement, and competing clinical demands, may inhibit learners from applying new behaviors in practice. Evaluating Level 3 and 4 outcomes also poses methodological challenges, as these require longitudinal follow-up and system-level measurement, which is rarely feasible in educational studies. To enhance long-term impact, MEERs could be embedded longitudinally within curricula, paired with reinforcement and debriefing activities, integrated into clinical training contexts, and aligned with institutional safety and quality priorities. These strategies may help bridge the gap between short-term learning gains and measurable improvements in professional behavior and patient outcomes.

This review also highlights the versatility of MEERs across a range of educational objectives: they were used to introduce new concepts, review material, reinforce clinical protocols, identify safety hazards, and foster team bonding. Interestingly, Lopex-Pernas *et al.* found that students with stronger subject familiarity reported greater perceived learning, while MEERs aimed at teaching entirely new material received more critical evaluations ^35^. This may reflect the cognitive load imposed by complex escape room tasks, which can overwhelm learners unfamiliar with the foundational material. Future instructional design should consider sequencing MEERs after initial exposure to content or pairing them with preparatory learning activities to optimize educational value.

Comparative studies further underscore the value of MEERs. When measured against flipped classrooms and traditional lectures, MEERs generally yielded greater knowledge gains and learner satisfaction, supporting their potential as highly engaging and effective instructional tools ^9, 26^. While only two comparative studies directly evaluated MEERs against these methods, both reported favorable outcomes, suggesting that integrating gamified and experiential elements may enhance critical thinking and long-term retention compared to conventional didactic approaches. These findings are consistent with educational theories that emphasize the benefits of active learning and learner immersion in realistic, problem-solving contexts ^27^. Although the limited number of comparative studies precludes definitive conclusions, the available evidence indicates that MEERs perform at least as well as, and often better than, traditional and active learning modalities, particularly in fostering engagement, motivation, and collaboration. Future comparative and meta-analytic studies are needed to confirm these preliminary trends and further delineate where MEERs offer the most significant educational advantage.

Despite their benefits, MEERs have practical limitations. They can be resource-intensive to design and implement, requiring substantial faculty time for planning, puzzle creation, facilitation, and debriefing. Physical space constraints, particularly in programs without dedicated simulation areas, can limit realism and scalability. Additionally, material costs for equipment, props, and maintenance can pose further barriers. These challenges can be mitigated through collaborative development, the use of low-cost or reusable materials, and adaptation to virtual or shared spaces to reduce time and financial demands. Time limitations may also lead to superficial coverage of complex topics, and poorly designed puzzles or uneven group dynamics can diminish educational value. Finally, MEERs may be less accessible for learners with disabilities, language barriers, or social anxiety, emphasizing the importance of inclusive design and flexible participation formats to ensure equitable learning experiences.

To our knowledge, this is the first scoping review to systematically evaluate the use of Medical Education Escape Rooms (MEERs) specifically within undergraduate and graduate medical education, using the Kirkpatrick Model as a structured framework to classify outcomes. While previous studies have explored gamification or individual escape room interventions more broadly, this review is novel in its exclusive focus on MEERs designed for medical students and residents, offering a targeted synthesis of their educational value in clinical training contexts. By categorizing findings across reaction, learning, behavior, and results, this study not only highlights the consistent benefits of MEERs in promoting engagement and knowledge acquisition but also identifies a gap in evidence regarding long-term behavioral and clinical impact.

Future research should include interprofessional learner groups, such as nursing, pharmacy, and allied health trainees, to better reflect real-world healthcare teams and expand the generalizability of findings. Incorporating diverse professional perspectives within MEERs may enhance collaboration, communication, and situational awareness, skills essential for patient safety and effective interprofessional practice. Such inclusion would also help evaluate how MEERs can facilitate shared learning and team-based problem solving across healthcare disciplines.

### Limitations

One limitation of this study is that it was restricted to studies involving medical students and residents, excluding other healthcare trainees such as dental, pharmacy, and nursing students. As a result, the number of included studies was limited, particularly those evaluating long-term behavioral outcomes. Future research should explore MEER use across a wider range of healthcare disciplines to better understand their broader applicability. There are also limitations in using the Kirkpatrick Model to evaluate educational interventions. Measuring Levels 3 (behavior) and 4 (organizational outcomes) is challenging, especially in medical education. For example, assessing whether participants apply skills in clinical settings requires long-term observation, and attributing behavioral change solely to training is difficult due to ongoing clinical learning. Organizational shifts, such as system updates, may further confound results. Evaluating Level 4 outcomes demands significant time, resources, and expertise.

Finally, the scoping review methodology has inherent constraints. While it provides a broad overview of the existing literature, it does not offer in-depth analysis or definitive conclusions. Moreover, this review did not include a formal critical appraisal of study quality, as is consistent with scoping review methodology. Nonetheless, several methodological limitations across included studies warrant acknowledgment. Many studies employed small sample sizes, lacked control groups, and relied heavily on self-reported outcomes, which may introduce bias and limit the generalizability of their findings. These factors should be considered when interpreting the overall strength of evidence regarding MEER effectiveness.

## Conclusion(s)

Escape rooms are a valuable and versatile educational tool for teaching clinical concepts, reinforcing skills, and enhancing learner well-being in medical education. They consistently generate positive reactions and improve clinical knowledge and confidence. MEERs have outperformed traditional lectures and flipped classrooms and are as effective as CBL sessions, suggesting they could serve as an alternative or supplement within training programs. Most MEERs were used for reviewing content or reinforcing existing knowledge, which represents their most effective application. Further research should examine the feasibility and resources required to scale MEERs as CBL alternatives. While current evidence does not demonstrate consistent behavioral change, longitudinal studies are needed to explore their long-term impact on clinical practice and professional development.

## Statements and declarations

### Ethical considerations

Ethical approval was not required for this study as it involved the synthesis of existing, publicly available data and did not include human participants.

## Data Availability

Data is publicly available on Zenodo under the DOI: 10.5281/zenodo.15649841 ^36^. Data are available under the terms of the Creative Commons Attribution 4.0 International

## Appendix

### Search Strategies

**PubMed:**

(("escape"[All Fields] OR "escaped"[All Fields] OR "escapement"[All Fields] OR "escapes"[All Fields] OR "escaping"[All Fields]) AND "room"[All Fields]) AND ("2010/01/01"[Date - Publication] : "2022/12/31"[Date - Publication])

**MedEdPORTAL:**

(("escape" OR "escaped" OR "escapement" OR "escapes" OR "escaping") AND "room") AND (publication_date:[2010 TO 2022])

**Scopus:**

(TITLE-ABS-KEY("escape" OR "escaped" OR "escapement" OR "escapes" OR "escaping") AND TITLE-ABS-KEY("room")) AND (PUBYEAR >= 2010 AND PUBYEAR <= 2022)
