# Supplementary material for: Evaluating Medical Education Escape Rooms: A Scoping Review Using the Kirpatrick Model
Source: MedEdPublish (2016). 2026 Jan 29;15:28. Originally published 2025 Jul 2. [Version 3] doi: 10.12688/mep.21095.3 (PMC12905535; doi:10.12688/mep.21095.3)
Supplement: Peer review history

# Reviewer report: Reviewer response for version 3

**DOI:** 10.21956/mep.23019.r45254

**Published:** 2026-02-13

**Authors:** Samar A Hegazy

**Affiliations:** 1 University of Illinois Urbana-Champaign, Urbana, USA

The current version of the manuscript addresses the outstanding points from the prior review. The search strategy has been clarified and made consistent across databases, and the Methods section now includes an explanation and citation for the effect-size calculations. These revisions improve methodological transparency.

 No further substantive comments remain at this stage. The manuscript is suitable for indexing.

Have any limitations of the research been acknowledged?

Yes

Is the work clearly and accurately presented and does it cite the current literature?

Yes

If applicable, is the statistical analysis and its interpretation appropriate?

Not applicable

Are all the source data underlying the results available to ensure full reproducibility?

Yes

Is the study design appropriate and is the work technically sound?

Partly

Are the conclusions drawn adequately supported by the results?

Yes

Are sufficient details of methods and analysis provided to allow replication by others?

Partly

Reviewer Expertise:

Medical Education Research, including review papers.

I confirm that I have read this submission and believe that I have an appropriate level of expertise to confirm that it is of an acceptable scientific standard.

# Reviewer report: Reviewer response for version 2

**DOI:** 10.21956/mep.22871.r44173

**Published:** 2026-01-10

**Authors:** Chaewon Kim

**Affiliations:** 1 Florida State University, Florida, USA

I think the authors have done a great job addressing the previous comments. I'm happy to see this article officially indexed.

Have any limitations of the research been acknowledged?

Partly

Is the work clearly and accurately presented and does it cite the current literature?

Yes

If applicable, is the statistical analysis and its interpretation appropriate?

Not applicable

Are all the source data underlying the results available to ensure full reproducibility?

Yes

Is the study design appropriate and is the work technically sound?

Yes

Are the conclusions drawn adequately supported by the results?

Partly

Are sufficient details of methods and analysis provided to allow replication by others?

Yes

Reviewer Expertise:

instructional design, game-based learning, nursing education

I confirm that I have read this submission and believe that I have an appropriate level of expertise to confirm that it is of an acceptable scientific standard.

# Reviewer report: Reviewer response for version 2

**DOI:** 10.21956/mep.22871.r44172

**Published:** 2025-11-22

**Authors:** Samar A Hegazy

**Affiliations:** 1 University of Illinois Urbana-Champaign, Urbana, USA

The revised manuscript has addressed nearly all prior comments thoroughly and thoughtfully. The authors strengthened methodological transparency by providing an appendix with search strings, registering their protocol, incorporating effect‐size estimates, expanding discussion of higher‐level outcomes, and adding interprofessional and resource considerations. The revisions clearly improve the paper’s rigor and educational relevance.

 I have only two minor points remaining for full transparency:

1. Search strategies:
  The appendix search strings currently differ across databases (e.g., PubMed includes filters such as *clinicaltrial*, *meta-analysis*, and a 10-year limit, while the Methods state inclusion of all study types over the past 15 years). The authors should clarify in the manuscript which search strategy was actually executed for each database—specifying real date ranges and filters used—and ensure consistency between the Methods and appendix. If the listed strings are illustrative rather than exact queries, that should be stated explicitly.
2. Effect-size methodology:
  Because Cohen’s *d* values were newly introduced, please include in the Methods a concise description of how these were derived (paired vs. pooled) and cite the methodological source(s) actually used for those calculations or approximations. No re-analysis is requested.

Once the search strategy is corrected and the methodological source(s) cited, the paper will meet full reporting standards.

Have any limitations of the research been acknowledged?

Yes

Is the work clearly and accurately presented and does it cite the current literature?

Yes

If applicable, is the statistical analysis and its interpretation appropriate?

Not applicable

Are all the source data underlying the results available to ensure full reproducibility?

Yes

Is the study design appropriate and is the work technically sound?

Partly

Are the conclusions drawn adequately supported by the results?

Yes

Are sufficient details of methods and analysis provided to allow replication by others?

Partly

Reviewer Expertise:

Medical Education Research, including review papers.

I confirm that I have read this submission and believe that I have an appropriate level of expertise to confirm that it is of an acceptable scientific standard, however I have significant reservations, as outlined above.

# Reviewer report: Reviewer response for version 1

**DOI:** 10.21956/mep.22598.r42574

**Published:** 2025-09-01

**Authors:** Samar A Hegazy

**Affiliations:** 1 University of Illinois Urbana-Champaign, Urbana, USA

**Manuscript Title:** *Evaluating Medical Education Escape Rooms: A Scoping Review Using the Kirkpatrick Model*

**Authors:** Sturm H, Brown G, Gantwerker EA

**Journal:** *MedEdPublish* (2025)

 Overall Assessment

 This manuscript presents a scoping review of Medical Education Escape Rooms (MEERs) in undergraduate and graduate medical education, analyzed through the Kirkpatrick model. It synthesizes 20 studies and offers insights into learner reactions, knowledge gains, and limited attempts at behavior change.

 The topic is timely, original, and of practical importance for medical educators. The study is well-structured, draws on current literature, and makes a meaningful contribution as the first scoping review explicitly focusing on MEERs in medical education.

 Evaluation Against Review Criteria

1. **Is the work clearly and accurately presented and does it cite the current literature?**
**Yes** – The paper is clearly written, well-structured, and grounded in contemporary and foundational sources (e.g., gamification, active learning, experiential learning). Literature is up to date through 2023–24.
2. **Is the study design appropriate and is the work technically sound?**
**Partly** – The scoping review framework (Arksey & O’Malley, JBI guidelines) is appropriate. However, the restriction to the single search term “escape room” may have excluded relevant variations (e.g., “breakout box”), and no critical appraisal of included studies was performed.
3. **Are sufficient details of methods and analysis provided to allow replication by others?**
**Partly** – The inclusion/exclusion criteria, database selection, and PRISMA flow are described, but full search strings and coding/analysis decisions are not provided. Replicability would be improved by publishing the protocol or appendix with detailed strategies.
4. **If applicable, is the statistical analysis and its interpretation appropriate?**
**Not applicable / Partly** – As a scoping review, the study appropriately employs descriptive synthesis rather than formal statistical analysis. Descriptive summaries (percentages, pre/post changes) are adequate, though effect size reporting where available could strengthen interpretation.
5. **Have any limitations of the research been acknowledged?**
**Yes** – The authors acknowledge limitations in scope (restricted to medical students/residents, exclusion of non-English studies), challenges in applying the Kirkpatrick model, and the inherent constraints of scoping reviews.
6. **Are all the source data underlying the results available to ensure full reproducibility?**
**Yes** – The authors provide open access to their extracted data on Zenodo.
7. **Are the conclusions drawn adequately supported by the results?**
**Yes** – Conclusions are measured and aligned with findings. The authors avoid overstatement, noting that MEERs demonstrate clear short-term benefits but limited evidence of behavioral or clinical impact.

Major Comments (must be addressed)

1. **Search Strategy Transparency**
  - Provide full search strings for each database in an appendix to ensure transparency and reproducibility.
  - Justify exclusion of terms such as *breakout box* or *puzzle-based learning*, which may have captured additional relevant studies.
2. **Quality Appraisal of Included Studies**
  - While scoping reviews do not always include critical appraisal, some commentary on the methodological rigor or risk of bias of included studies would strengthen the validity of conclusions.
3. **Analysis of Learning Outcomes**
  - Where available, extract and report effect sizes or standardized mean differences from pre/post-test studies. This would allow a stronger statement about learning gains.
4. **Discussion of Higher-Level Outcomes**
  - Expand the discussion on why MEERs do not consistently translate to behavior change (Kirkpatrick Level 3) or organizational outcomes (Level 4). Suggest curricular strategies (e.g., longitudinal embedding, reinforcement activities, integration with clinical practice) that could enhance long-term impact.

Minor Comments (suggested improvements)

1. Clarify whether the review protocol was registered (e.g., Open Science Framework, PROSPERO equivalent for education).
2. Tables summarizing study outcomes are useful; however, consider a consolidated summary highlighting effect sizes, sample sizes, and design type for ease of comparison.
3. Expand on the resource implications of MEERs (cost, faculty time, space) in the discussion to aid institutions considering adoption.
4. Future research directions should explicitly recommend inclusion of interprofessional groups (nursing, pharmacy, allied health) to broaden applicability.

Recommendation

 The manuscript is **original, relevant, and potentially impactful**. With **revisions to strengthen transparency, methodological rigor, and the discussion of long-term outcomes**, it will be scientifically sound and highly valuable for medical educators.

Have any limitations of the research been acknowledged?

Yes

Is the work clearly and accurately presented and does it cite the current literature?

Yes

If applicable, is the statistical analysis and its interpretation appropriate?

Not applicable

Are all the source data underlying the results available to ensure full reproducibility?

Yes

Is the study design appropriate and is the work technically sound?

Partly

Are the conclusions drawn adequately supported by the results?

Yes

Are sufficient details of methods and analysis provided to allow replication by others?

Partly

Reviewer Expertise:

Medical Education Research, including review papers.

I confirm that I have read this submission and believe that I have an appropriate level of expertise to confirm that it is of an acceptable scientific standard, however I have significant reservations, as outlined above.

# Response

**Published:** 2025-10-06

**Authors:** Hannah Sturm

Major Comments (must be addressed)

1. **Search Strategy Transparency**
  - **Provide full search strings for each database in an appendix to ensure transparency and reproducibility.**
  - **Justify exclusion of terms such as *breakout box *or *puzzle-based learning*, which may have captured additional relevant studies.**

We appreciate the reviewer’s emphasis on search transparency. We have added an appendix containing the complete search strings for each database (PubMed, MedEdPORTAL, and Scopus) (pages 13-14, lines 525-536). In the Methods section, we now provide a detailed rationale for excluding broader terms, such as “ *breakout box”* *and “* *puzzle-based learning” (pages 4-5, lines 157-162)* *.* These terms were intentionally omitted because preliminary scoping and test searches demonstrated that they frequently retrieved non-educational or recreational results unrelated to structured health professions education interventions. Moreover, terminology describing educational escape rooms has become standardized in recent literature, with “escape room” consistently used to refer to this instructional format. Including broader or ambiguous terms would likely have reduced the precision of the search without meaningfully improving sensitivity. Therefore, we employed a focused search strategy using the single keyword “escape room” to balance between comprehensiveness and relevance.  

1. **Quality Appraisal of Included Studies**
  - **While scoping reviews do not always include critical appraisal, some commentary on the methodological rigor or risk of bias of included studies would strengthen the validity of conclusions.**

Thank you for this valuable suggestion. Although formal quality appraisal is not required in scoping reviews, we agree that addressing study quality enhances the interpretability of the results. We have added a brief subsection to the limitations section that describes the methodological rigor and potential biases of the included studies. This commentary summarizes common limitations, including small sample sizes, the lack of control groups, and reliance on self-reported outcomes, and discusses how these factors may temper conclusions about MEER effectiveness (pages 10-11, lines 395-401).  

1. **Analysis of Learning Outcomes**
  - **Where available, extract and report effect sizes or standardized mean differences from pre/post-test studies. This would allow a stronger statement about learning gains.**

We thank the reviewer for this excellent suggestion to strengthen the analysis of learning outcomes. In response, we extracted and reported Cohen’s *d* effect sizes for studies that included pre- and post-test data. Where standard deviations (SD) were available, *d* values were calculated directly using pooled or paired SDs. Where only ranges or *p*-values were reported, approximate effect sizes were estimated using established methods (e.g., range-based or pooled-SD approximations). These values have been integrated into the Results section to quantify the magnitude of learning gains (page 7, lines 256-269).  

1. **Discussion of Higher-Level Outcomes**
  - **Expand the discussion on why MEERs do not consistently translate to behavior change (Kirkpatrick Level 3) or organizational outcomes (Level 4). Suggest curricular strategies (e.g., longitudinal embedding, reinforcement activities, integration with clinical practice) that could enhance long-term impact.**

We thank the reviewer for this valuable suggestion. The Discussion has been expanded to address why MEERs did not show measurable outcomes at Kirkpatrick Level 3, highlighting factors such as limited longitudinal exposure, lack of reinforcement, and challenges in assessing behavior change in clinical settings. We also added strategies to enhance long-term impact, including longitudinal embedding, reinforcement activities, integration with clinical practice and debriefing, and alignment with institutional safety and quality goals (page 9, lines 327–341).

 Minor Comments (suggested improvements)

1. **Clarify whether the review protocol was registered (e.g., Open Science Framework, PROSPERO equivalent for education).**

Thank you for this comment. The review protocol has now been registered on Open Science Framework. This has also been added to the methods section for clarification (page 4, lines 142-144).  

1. **Tables summarizing study outcomes are useful; however, consider a consolidated summary highlighting effect sizes, sample sizes, and design type for ease of comparison.**

We thank the reviewer for this helpful suggestion. In response, we created a **consolidated summary table** that presents key study characteristics, including **sample sizes, study designs,** **outcomes,** **and estimated effect sizes**, to facilitate clearer cross-study comparison of outcomes. All the data has been consolidated into Table 1 and referenced in the text to enhance readability and synthesis of findings.  

1. **Expand on the resource implications of MEERs (cost, faculty time, space) in the discussion to aid institutions considering adoption.**

We appreciate the reviewer’s thoughtful suggestion. In response, we have expanded the Discussion to include a brief analysis of the practical and resource-related considerations associated with implementing MEERs. The revised text now addresses financial and logistical factors, such as material costs, faculty preparation time, and space constraints, and provides context for how institutions can adapt MEERs within their existing resources through creative and scalable approaches (pages 9-10, lines 352-362).  

1. **Future research directions should explicitly recommend inclusion of interprofessional groups (nursing, pharmacy, allied health) to broaden applicability**

We appreciate this insightful recommendation. In response, we have revised the **Discussion** section to emphasize the importance of including **interprofessional learner groups**, such as nursing, pharmacy, and allied health trainees, in future MEER studies. This addition highlights how interprofessional participation can enhance teamwork, communication, and realism, thereby broadening the applicability of MEERs across healthcare education contexts (page 10, lines 373-379).

# Reviewer report: Reviewer response for version 1

**DOI:** 10.21956/mep.22598.r42467

**Published:** 2025-08-30

**Authors:** Chaewon Kim

**Affiliations:** 1 Florida State University, Florida, USA

1. Why was the Kirkpatrick model chosen as a framework for this study when the level 3 and 4 is not really examined?

 2. Clearer definition of serious games and gamification and how the types of escape rooms based on each approach differ should be provided in more details.

 3. "Only one study assessed Kirkpatrick Level 3 outcomes, which evaluate behavioral change. This study aimed to improve residents' understanding of patient safety priorities and voluntary event reporting systems. Six months after the intervention, there was no significant increase in the use of the reporting system compared to historical controls, indicating no measurable behavioral change." - Which study?

 4. How is MEER different from EERs in other domains? 

 5. There were only two comparative studies that compared EER to CBL or flipped classrooms. It should be cautious to claim that EER outperfoms those approaches based on such little evidence, more so because this is not a meta-analysis.

 6. I suspect the body text is partially written by generative AI, especially when the MEER is restated in its full name in the second paragraph of the discussion. Using AI-generated text directly in the manuscript is unethical.

Have any limitations of the research been acknowledged?

Partly

Is the work clearly and accurately presented and does it cite the current literature?

Yes

If applicable, is the statistical analysis and its interpretation appropriate?

Not applicable

Are all the source data underlying the results available to ensure full reproducibility?

Yes

Is the study design appropriate and is the work technically sound?

Yes

Are the conclusions drawn adequately supported by the results?

Partly

Are sufficient details of methods and analysis provided to allow replication by others?

Yes

Reviewer Expertise:

instructional design, game-based learning, nursing education

I confirm that I have read this submission and believe that I have an appropriate level of expertise to state that I do not consider it to be of an acceptable scientific standard, for reasons outlined above.

# Response

**Published:** 2025-10-06

**Authors:** Hannah Sturm

**Responses to Reviewer 1**

1. **Why was the Kirkpatrick model chosen as a framework for this study when the level 3 and 4 is not really examined?**

We appreciate the reviewer’s observation regarding the use of the Kirkpatrick model. The model was chosen because it is one of the most widely used frameworks in medical education to evaluate training interventions and allows for comparisons across diverse instructional approaches. While most included studies were limited to Levels 1 and 2, adopting the framework provided a structured lens to highlight the paucity of research at Levels 3 and 4. We have revised the methods section to make this rationale explicit and to clarify that the model was applied primarily as an organizing framework, rather than implying comprehensive coverage across all levels (page 5, lines 187-192).

**2. Clearer definition of serious games and gamification and how the types of escape rooms based on each approach differ should be provided in more details.**

 We appreciate this suggestion and have expanded the introduction to provide clearer, literature-based definitions of “serious games” and “gamification.” We have also clarified how MEERs can be designed using either approach, using more specific examples of the integration of each technique into escape room design (page 3, lines 93-101).  

**3. "Only one study assessed Kirkpatrick Level 3 outcomes, which evaluate behavioral change. This study aimed to improve residents' understanding of patient safety priorities and voluntary event reporting systems. Six months after the intervention, there was no significant increase in the use of the reporting system compared to historical controls, indicating no measurable behavioral change." - Which study?**

 Thank you for pointing out this missing citation. We have now cited the specific study that assessed Kirkpatrick Level 3 outcomes (focusing on patient safety priorities and voluntary event reporting), ensuring the reader can locate and evaluate the original work (page 8, line 288).

**4. How is MEER different from EERs in other domains?**

 We agree that the distinction should be more clearly articulated. We have added a paragraph in the Introduction outlining how MEERs differ from EERs in other disciplines (page 3, lines 102-110).

**5. There were only two comparative studies that compared EER to CBL or flipped classrooms. It should be cautious to claim that EER outperfoms those approaches based on such little evidence, more so because this is not a meta-analysis.**

 We appreciate this important clarification. The statement has been revised to reflect that while some comparative studies suggest favorable outcomes for MEERs relative to traditional approaches, the evidence base remains limited, and conclusions should be interpreted with caution (pages 8-9, lines 316-328).

**6. I suspect the body text is partially written by generative AI, especially when the MEER is restated in its full name in the second paragraph of the discussion. Using AI-generated text directly in the manuscript is unethical.**

 We take this concern very seriously and wish to clarify. Generative AI was **not used to draft or** **write the content** of the manuscript. In line with journal policy, AI tools were employed solely **for grammar and spelling checking**, which falls under the journal’s rule that *“disclosure is not required for basic AI tools used solely for grammar, spelling, or reference checking.”*   The manuscript text itself was written entirely by the authors. To avoid any appearance of redundancy that may have led to this concern, we will revise the discussion section to streamline terminology and phrasing.
